# Combination of body mass index and oxidized low density lipoprotein receptor 1 in prognosis prediction of patients with squamous non-small cell lung cancer

**DOI:** 10.18632/oncotarget.4299

**Published:** 2015-05-27

**Authors:** Long Jiang, Shanshan Jiang, Yongbin Lin, Han Yang, Zerui Zhao, Zehua Xie, Yaobin Lin, Hao Long

**Affiliations:** ^1^ Sun Yat-Sen University Cancer Center, State Key Laboratory of Oncology in South China, Collaborative Innovation Center for Cancer Medicine, Guangzhou, China; ^2^ Lung Cancer Institute of Sun Yat-Sen University, Guangzhou, China; ^3^ Department of Thoracic Oncology, Sun Yat-Sen University Cancer Center, Guangzhou, China; ^4^ University of California, San Francisco, San Francisco, USA

**Keywords:** squamous non-small cell lung cancer, oxidized low density lipoprotein receptor 1, body mass index, prediction model

## Abstract

Lung cancer, especially non-small cell lung cancer (NSCLC), represents enormous challenges in continuously achieving treatment improvements. Besides cancer, obesity is becoming ever more prevalent. Obesity is increasingly acknowledged as a major risk factor for several types of common cancers. Significant mechanisms overlap in the pathobiology of obesity and tumorigenesis. One of these mechanisms involves oxidized low density lipoprotein receptor 1 (OLR1), as a link between obesity and cancer. Additionally, body mass index (BMI) has been widely used in exploiting the role of obesity on a series of diseases, including cancer. Significantly, squamous NSCLC revealed to be divergent clinical and molecular phenotypes compared with non-squamous NSCLC. Consequently, OLR1 immunostaining score and BMI were assessed by Fisher's linear discriminant analysis to discriminate if progression-free survival (PFS) would exceed 2 years. In addition, the final model was utilized to calculate the discriminant score in each study participant. Finally, 131 patients with squamous NCSLC were eligible for analysis. And a prediction model was established for PFS based on these 2 markers and validated in a second set of squamous NCSLC patients. The model offers a novel tool for survival prediction and could establish a framework for future individualized therapy for patients with squamous NCSLC.

## INTRODUCTION

Lung cancer, with an increasing incidence, is the leading cause of cancer-related death worldwide [1]. Therein, non-small cell lung cancer (NSCLC) accounts for 80% of lung cancers [2]. Under multimodality treatment, modest improvements in the survival rates of NSCLC have been reported [3]. Besides increasing incidence of cancer, obesity is becoming more and more prevalent in most developed countries in the recent decades. Moreover, worldwide obesity epidemic showed no signs of abating, although obesity-induced metabolic syndromes was ameliorated because of weight loss though exercise or dietary control [4]. Obesity was recognized a harmful effects on human health by Hippocrates, named as the father of medicine [5]. 2,000 years later, Robert Thomas established the first link between obesity and endometrial cancer [6]. After that, obesity is increasingly recognized as a major risk factor for several types of common cancers [7]. American Cancer Society concluded a higher cancer-related death rate in obese cohort than normal weight individuals [8]. Indeed, approximate 20 % cancer-related death was estimated to be caused by excess weight [9]. The higher rate of cancer deaths in obese population might account for the enhancing effects of obesity on cancer potency and progression [10]. Increased incidence and aggressiveness of tumor formation occurred in patients with obesity [11]. On the other hand, evidences also illustrated that surgical (bariatric surgery) or dietary weight-losing interventions could reduce the risk of cancers [12].

The above phenomenon suggested a mechanistic overlap in the pathobiology of obesity and tumorigenesis [13]. Oxidized low density lipoprotein receptor 1 (OLR1), highly conserved in mammals, is a lectin-like scavenger receptor, which could recognize several ligands, such as protein moiety of oxidized- low- density lipoprotein (LDL) [14]. Recently, OLR1 has indicated as link between obesity and cancer [15]. OLR1 could be activated through NF-kB activated inflammatory signaling, a strongly implicated signaling in carcinogenesis [15].

Nowadays, treatment failures still represent enormous challenges and it is doubtful if these standard treatment modalities could continuously achieve substantial improvements [16]. Additionally, squamous NSCLC revealed to be divergent clinical and molecular phenotypes compared with non-squamous NSCLC [17]. Therefore, novel characters in Squamous NSCLC is hoped to be explored and confirmed to develop relative approach in managing squamous NSCLC [18]. Body mass index (BMI), as a surrogate marker of general adiposity, has been widely used in exploiting the role of obesity on a series of diseases, such as cardiovascular disease, type 2 diabetes, osteoarthritis, as well as cancer [19]. In fact, epidemiological studies have shown an elevated risk of several types of cancer in population with excess BMI [20-23]. In the meantime, application of BMI allowed exploring relationship between normal weight, overweight and obesity, and the risk of cancer [22]. Nevertheless, the effect of increased BMI on cancer incidence was not equally extensive to all types of cancer [24]. Thus, respective assessment remains necessary [5]. Besides, the prognostic value of BMI is still under debate, excess BMI not always associating with poor prognosis [8, 25].

Significantly, combining application of appropriate biomarkers in prognosis prediction is emerging its high importance in cancer research [26]. Consequently, we evaluate the prognostic effect of combining BMI and OLR1 in patients with squamous NSCLC, which has not been combining used in other types of cancer. A prediction model for progression-free survival (PFS) was derived based on combining BMI and OLR1 and further validated in a second set of squamous NCSLC patients.

## RESULTS

### Clinical outcomes

Totally, 131 patients with squamous NCSLC were eligible for the final analysis. The mean age was 59.56 years (range: 32–80 years, median 60 years); 116 patients were male (88.5%) and 15 female (11.5%). 103 (78.6%) patients were smokers. Moreover, stage IA disease occurred in 19 (14.5%) patients, IB in 36 (27.5%), IIA in 17 (13.0%), IIB in 17 (13.0%), IIIA in 40 (30.5%), and IIIB in 2 (1.5%). In addition, locations of tumor were 37 (28.2%) in left upper lobe, 27 (20.6%) in left lower lobe, 27 (20.6%) in right upper lobe, 11 (8.4%) in right middle lobe, and 29 (22.1%) in right lower lobe. Meantime, pathological analysis reported 7 patients (5.3%) with well differentiated, 36 (27.5%) with moderately differentiated, and 88 (67.2%) with poorly differentiated.

**Figure 1:**
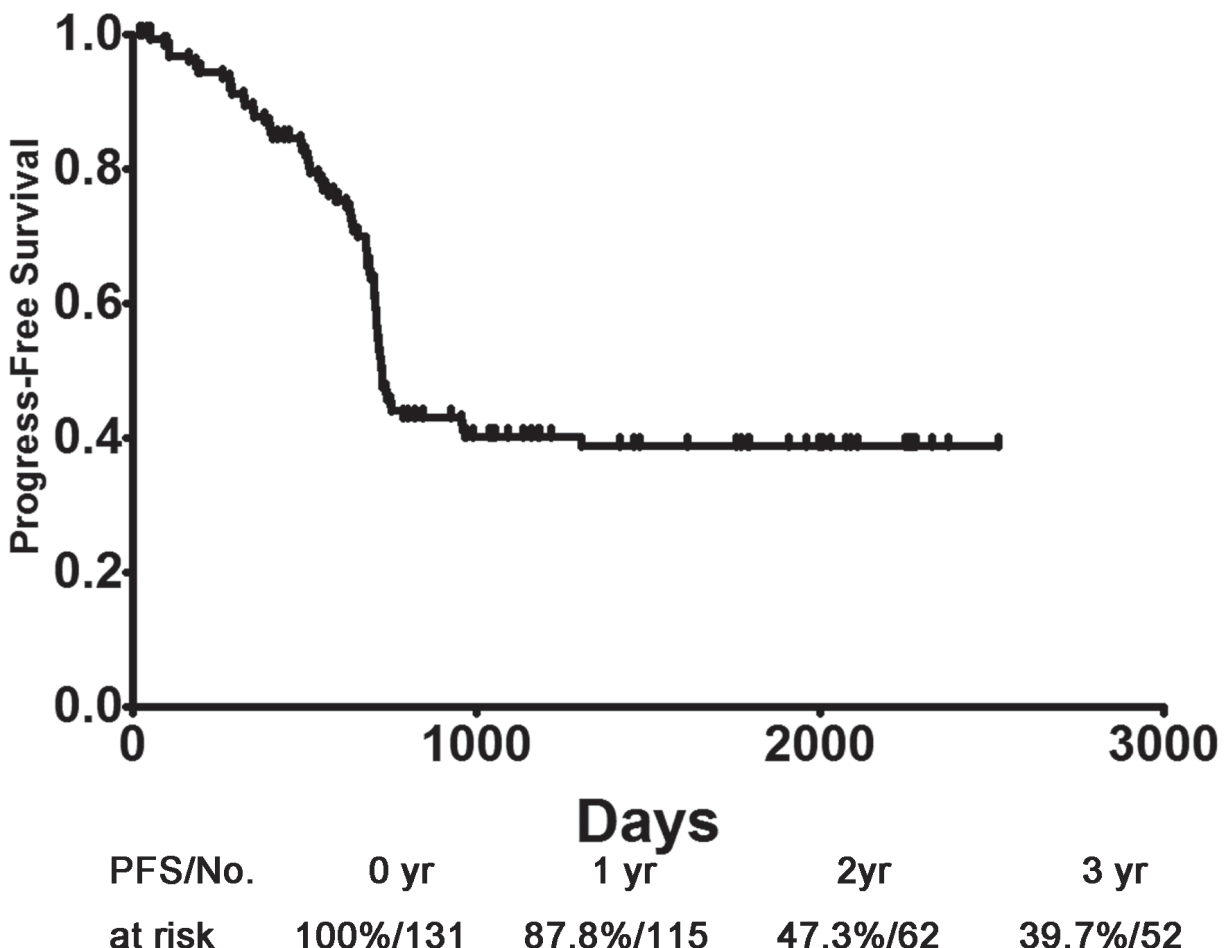
PFS of patients with squamous non-small cell lung cancer PFS: progression-free survival.

The mean follow-up for survivors as of December 2014 was 47.23 months (range: 0.63–90.83 months, median 49.03 months). Furthermore, mean PFS, due to last follow-up, was 724 days and the overall 1-, 2- and 3-year PFS rates were 87.8%, 47.3% and 39.7%, respectively (Figure 1). Univariate and multivariate analysis proved the prognostic role of OLR1 and BMI for PFS, both separately and together. (Supplemental Table S1).

**Figure 2:**
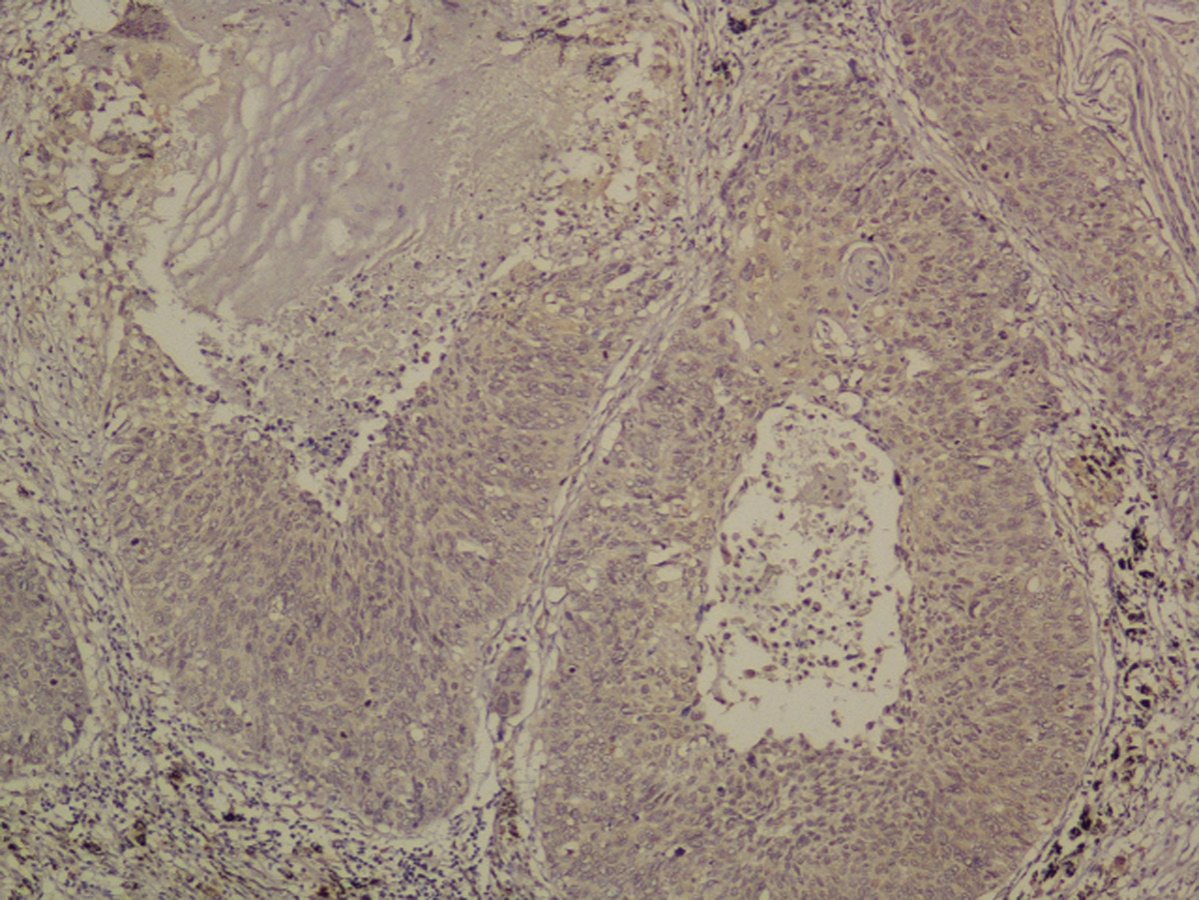
Immunohistochemistry for OLR1 Original magnification 200x.

OLR1 expressed on tumor cells. Among all these 131 specimens of squamous NCSLC, OLR1 expressed in either one or both of the cell membrane and cytoplasm, in a focal or scattered pattern (Figure 2).

There was no significant difference between the training (*n* = 87) and validation (*n* = 44) cohorts in patients' sex, age, smoking habit, tumor size, tumor location, differentiation, pathological stage, follow-up, OLR1 immunostaining score (*P* > 0.1) (Table 1).

**Table 1:** Clinicopathological characters in training and validation cohorts

Characteristic	All (n = 131)		Training cohorts (n = 87)	Validation cohorts (n = 44)	*P*
Age, yrs	60^†^ (range: 32–80)		61^†^ (range: 32–80)	58^†^ (range: 32–75)	0.590
Sex (%)					0.546
Male	116	88.5%	76	40	
Female	15	11.5%	11	4	
Smoking habit					0.278
No	28	21.4%	21	7	
Yes	103	78.6%	66	37	
Tumor Size (cm)	4 ^†^ (range: 0.6–11)		4^†^ (range: 1–8)	4.5^†^ (range: 0.6–11)	0.672
Tumor location					0.689
Left Upper Lobe	37	28.2%	27	10	
Left Lower Lobe	27	20.6%	15	12	
Right Upper Lobe	27	20.6%	16	11	
Right Middle Lobe	11	8.4%	7	4	
Right Lower Lobe	29	22.1%	22	7	
Tumor differentiation					0.379
Well differentiated	7	5.3%	6	1	
Moderately differentiated	36	27.5%	24	12	
Poorly differentiated	88	67.2%	57	31	
Pathological Stage (%)					0.878
IA	19	14.5%	12	7	
IB	36	27.5%	26	10	
IIA	17	13.0%	11	6	
IIB	17	13.0%	10	7	
IIIA	40	30.5%	26	14	
IIIB	2	1.5%	2	0	
Follow-up (months)					0.517
Median	49.03		48.70	51.75	
Range	0.63–90.83		0.63-90.83	1.37-83.90	
Mean	47.23		46.26	49.15	
OLR1 immunostaining score					0.135
0	18	13.7%	14	4	
1	28	21.4%	14	14	
2	39	29.8%	29	10	
3	46	35.1%	30	16	
BMI ( kg/m^2^)					0.209
Median	22.06		22.32	21.77	
Range	13.70-31.25		13.70-31.25	16.44-26.95	
Mean	22.26		22.48	21.82	

† Median values are listed

### Class prediction analysis

Based on training cohorts, BMI and OLR1 immunostaining score were used in setting a prediction model by employing Fisher's linear discriminant analysis (FLDA) with stepwise variant-selection. The clinical classifying model was described by the following equation: Y = −5.811 + 1.285 ×OLR1 immunostaining score + 0.152 ×BMI (eigenvalue 1.272, canonical correlation 0.748, *P* < 0.001).

**Figure 3:**
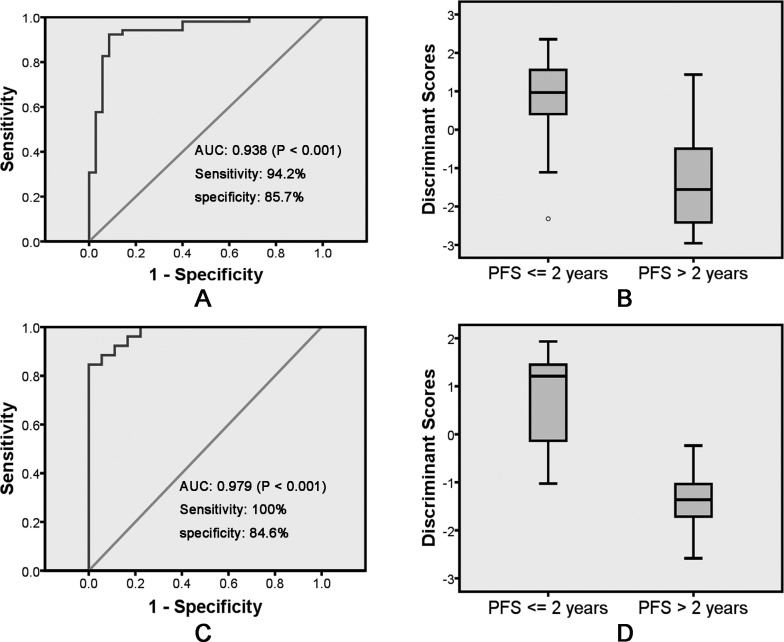
Receiver operating characteristic curve analysis of the discriminant model with BMI and OLR1 immunostaining score for discriminate PFS <= 2 years and PFS > 2 years on training **A.** and validation **C.** samples. Box and Whisker plot showing the distributions of the discriminant scores of PFS <= 2 years and PFS > 2 years in training **B.** and validation **D.** samples. PFS: progression-free survival.

Group centroids for PFS <= 2 years and PFS > 2 years were 0.914 and - 1.359, respectively. Next, a cut score halfway between the two centroids was determined: cut score= (−1.359 + 0.914)/2 = −0.2225. When the discriminant score Y was calculated to be > −0.2225, the case was predicted to be a PFS <= 2 years case; otherwise, the case was classified as a PFS > 2 years. For the training set of 87 leave-one-out-cross-validated cases, 49 of 52 PFS > 2 years (94.2% sensitivity) and 30 of 35 PFS <= 2 years (85.7% specificity) were correctly classified with an overall accuracy of 90.8% (79 of 87) and an area under the curve (AUC) of 0.938 [*P* < 0.001, 95% confidence interval (CI) 0.884 –; 0.993] (Table 2, Figure 3A and 3B).

**Table 2:** Distribution of actual and predicted progression-free survival of patients with lung squamous cell carcinoma

		Predicted results					
		Training phase ^a^			Validation phase		
		PFS > 2 years, n (%)	PFS <= 2 years, n (%)	Total, n	PFS > 2 years, n (%)	PFS <= 2 years, n (%)	Total, n
Actual results	PFS > 2 years, n (%)	49 (94.2) ^b^	3 (5.8)	52	18 (100) ^d^	0 (0)	18
	PFS <= 2 years, n (%)	5 (14.3)	30 (85.7) ^c^	35	4 (15.4)	22 (84.6) ^e^	26
Total, n		54	33	87	22	22	44

a Leave-one-out cross-validated grouped cases.

b, c The sensitivity ^b^ and specificity ^c^ for identifying PFS > 2 years were 94.2% and 85.7% for the training sets of leave-one-out cross-validated grouped cases, respectively.

d, e The sensitivity ^d^ and specificity ^e^ for identifying PFS > 2 years were 100% and 84.6% for the validation cases, respectively.

Next, the predicting model consisting of the 2 predictors (BMI and OLR1 immunostaining score) were applied to the validation set of 44 patients (18 PFS > 2 years and 26 PFS <= 2 years) (Table 2). A survival prediction for 40 of the 44 patients (90.9%) with an AUC of 0.979 (*P* < 0.001, 95% CI 0.806–1) was achieved (Table 2, Figure 3C and 3D). Also, 18 of 18 PFS > 2 years (100% sensitivity) and 22 of 26 PFS <= 2 years (84.6% specificity) were correctly identified (Table 2).

## DISCUSSION

Clinical and epidemiological evidences have indicated correlations between cancer and metabolic disorders. Specifically, high cancer incidence could be observed in obesity population [8, 27]. This correlation between cancer and obesity was robust, due to their sharing with common or similar molecular properties and biological programs, which led to common transcriptional signatures for a diverse set of diseases [28]. As a consequence, some drugs used in non-cancer diseases showed ability in inhibiting cellular transformation [15]. Previous studies demonstrated potential interconnected mechanisms involving excess adiposity and cancer risk, including insulin/insulin-like growth factor, circulating adipokines and systemic inflammatory mediators, sex steroids, and so on [5]. In addition, senescence-like features provoked by obesity would promote tumorigenesis. A well-studied example was senescence-associated secretory phenotype stimulating cancer development in both obese patients and mice [29]. Moreover, clinical trials proved both dietary and surgical weight loss interventions resulting in remarkable risk reductions in cancer [30]. In contrast with cancer incidence, obese or overweight patients were not always associated with poorer prognosis considering different cancer types [31, 32].

High BMI has been shown as a risk factor for a variety of high-risk diseases in previous studies, including cancer [33]. A number of meta-analyses, digging relative data from numerous prospective studies, established high BMI as obvious risk indicators of hepatocellular [34], gallbladder [35], pancreatic [36], gastric [23], rectal [37] and colon [38] cancers. Interestingly, meta-analyses indicated that the effect of high BMI on cancer risk is histological specificity [23]. One example is high BMI only decrease survival of esophageal adenocarcinoma patients but not esophageal squamous cell carcinoma [39].

As a lipid-related gene, OLR1 has been proved as a common hub between metabolism and cancer gene networks [40]. Strikingly, transformation of MCF-10A cells lacking ER-Src could be caused by oxidized LDL in a manner depending on NF-kB [15]. OLR1, as a receptor of oxidized LDL, is a marker for atherosclerosis, which could activate pathways involving inflammatory and hypoxia in macrophages and vascular endothelial cells [41]. Remarkably, OLR1 is critical in maintaining the transformation and growth states of cancer cell lines in diverse origins [42]. This phenomenon observed in xenografts experiments indicated the importance of OLR1 in connection between cancer and metabolic disorders [43]. Studies also suggested multiple potential associations between OLR1 and cancer susceptibility, such as OLR1 over-expression in human cancer cell line associated with obvious upregulation of several oncogenes and significant increase in cell apoptosis, proliferation and migration [15]. In addition, OLR1 took part in the whole technological procedure of de novo lipogenesis, on which many cancers exclusively rely regardless of nutritional availability [44]. The de novo lipogenesis procedure occurs early as a prerequisite for efficient transformation. This novel OLR1 procedure could account for much reported oncogenic activity, including transformation of epithelial cells, proliferation, migration, tumor growth and apoptosis [45].

Several limitations remain in this study. First, all the data were retrospectively collected, thus clinical and survival comparison might be influenced by selection bias due to its retrospective nature. Second, as a pilot exploratory study for squamous NCSLC, all patients were recruited from single institute, which means inevitable bias compared with real-world situation. However, separated training and validation set were used in developing and validating this prediction model. Additionally, result indicated sufficient predictive effect for further prospective validation in larger independent cohorts. Third, only 2 relative metabolic markers were used in this model. Although other metabolic markers known as prognostic factors was absent in current model, this concise prediction model offered satisfying predictive effect with convenient application.

In conclusion, our analyses demonstrated that the analysis of combination of BMI and OLR1 could effectively and reproducibly classify patients with squamous NCSLC according to their PFS. Further prospective validation in larger independent cohorts of patients with similar or different regimens is warranted to fully assess its predictive power. Moreover, together with widely application of positron emission tomography scan in malignances, combining these novel metabolic markers and imaging technologies could be employed in the whole process management of patients with malignancy. However, the combinational model offers a novel tool for survival prediction and could have important clinical implications for the consideration of differential treatment strategies in patients with squamous NCSLC, thus providing a framework for future individualized therapy.

## PATIENTS AND METHODS

Study protocol was approved by the Ethics Committee of Human Experimentation in China. Written informed consent was obtained from each patient: including signed consent for tissue analysis as well as consent to be recorded for potential medical research at the time of sample acquisition. All experiments were performed in accordance with relevant guidelines and regulations.

Chart review was performed on 1286 consecutive patients who suffered from squamous NSCLC with between November 2004 and March 2008. 131 of the 1286 patients were enrolled in the final analysis, while other patients with squamous NSCLC were excluded from analysis because of incomplete clinical or pathological data, such as unavailable formalin-fixed paraffin-embedded blocks. These 131 patients were randomly assigned (2:1) centrally by computer into training group (*n* = 87) and validation group (*n* = 44).

Characteristics of patients and tumors were collected. The weight and height were recorded the time of admission for all patients as routine clinical practice in SYSUCC. BMI was calculated as follow: BMI (kg/m^2^) = weight (kg)/height (m^2^). Surgically resected or biopsied specimens were fixed in formalin and embedded in paraffin for routine histopathological diagnosis and immunohistochemical analysis. Then, PFS was defined as the time from the first documentation to the time of tumor progression or death. Notably, all data were reviewed and confirmed by two independent pathologists based on WHO classification of lung cancer [46].

### Immunohistochemistry

Isolated tumors were fixed in 10% neutral buffered formalin for 48 h and embedded in paraffin according to standard protocols. Sections (thickness, 4μm) were deparaffinized and rehydrated in a graded series of alcohol solutions. For antigen retrieval, slides were immersed in ethylenediamine tetra-acetic acid (1 mmol/L, pH8.0) and boiled for 15 min in a microwave oven. Endogenous peroxidase activity was blocked in 3% H_2_O_2_ at room temperature for 15 min, and non-specific binding was abolished by 5% bovine serum albumin for 30 min. Sections were then stained with anti- OLR1 (rabbit anti- OLR1 polyclonal antibody; 1:100 dilution; Protein Tech, Shanghai, China) antibody at 4°C overnight. After washing with phosphate-buffered saline (PBS), sections were incubated with horseradish peroxidase -conjugated secondary antibody (Envision Detection kit, GK500705, Gene Tech, Shanghai, China) at room temperature for 30 min. After washing thrice with PBS, antibody complexes were colored with 3, 3′ -diamino benzidine and then counterstained with hematoxylin. Slides were dehydrated and evaluated.

### Semi-quantitative method

The total OLR1 immunostaining score was calculated as the sum of the positively stained tumor cells and staining intensity. Briefly, the percentage of positive staining was scored as “0” (<5%, negative), “1” (5–25%, sporadic), “2” (25–50%, focal), or “3” (>50%, diffuse). Staining intensity was scored as “0” (no staining), “1” (weak staining), “2” (moderate staining), or “3” (strong staining). Both the percentage of positive cells and the staining intensity were evaluated under double-blind conditions. The total immunostaining score was calculated as the value of percent positivity score × staining intensity score, and ranged from 0 to 9. We defined OLR1 expression levels as: “0” (score 0–1), “1” (2–3), “2” (4–6) and “3” (>6). The score assessment was performed independently by two independent pathologists blinded to the clinical parameters.

### Statistical analysis

The data are presented as the number (%) or median (range) unless otherwise stated. The Pearson χ^2^ test and Fisher's exact test were used for categorical data, and an independent sample t-test or the Mann-Whitney U test were used for numerical data. Prognostic role of OLR1 and BMI for PFS were assessed by univariate analysis with log rank test and multivariate analysis with Cox proportional hazards regression.

First, the final model was used to calculate the discriminant score in each study participant. Second, the comparison between the discriminant score with the PFS was used to construct a receiver operating characteristic curve. In the meantime, the AUC and its 95% CI were also reported to describe the accuracy of the model for identifying metastases in our study participants. And the eigenvalue and canonical correlation was used to evaluate model fit (*P* <0.05 was considered statistically significant). We internally validated the model using a cross-validation procedure, which enabled us to use the full data set for model development. *P* values <0.05 were considered statistically significant. Data analysis was performed using Predictive Analytics Software (PASW) Statistics 18.0 for Windows (SPSS Inc, Chicago, IL).

## SUPPLEMENTARY MATERIAL TABLE



## References

[1] Siegel RL, Miller KD, Jemal A (2015). Cancer statistics, 2015. CA Cancer J Clin.

[2] Devesa SS, Bray F, Vizcaino AP, Parkin DM (2005). International lung cancer trends by histologic type: male:female differences diminishing and adenocarcinoma rates rising. Int J Cancer.

[3] Molina JR, Yang P, Cassivi SD, Schild SE, Adjei AA (2008). Non-small cell lung cancer: epidemiology, risk factors, treatment, and survivorship. Mayo Clin Proc.

[4] Sun B, Karin M (2012). Obesity, inflammation, and liver cancer. J Hepatol.

[5] Calle EE, Kaaks R (2004). Overweight, obesity and cancer: epidemiological evidence and proposed mechanisms. Nat Rev Cancer.

[6] Demark-Wahnefried W, Platz EA, Ligibel JA, Blair CK, Courneya KS, Meyerhardt JA, Ganz PA, Rock CL, Schmitz KH, Wadden T, Philip EJ, Wolfe B, Gapstur SM, Ballard-Barbash R, McTiernan A, Minasian L (2012). The role of obesity in cancer survival and recurrence. Cancer Epidemiol Biomarkers Prev.

[7] Khandekar MJ, Cohen P, Spiegelman BM (2011). Molecular mechanisms of cancer development in obesity. Nat Rev Cancer.

[8] Calle EE, Rodriguez C, Walker-Thurmond K, Thun MJ (2003). Overweight, obesity, and mortality from cancer in a prospectively studied cohort of U.S. adults. N Engl J Med.

[9] Wolin KY, Carson K, Colditz GA (2010). Obesity and cancer. Oncologist.

[10] Reeves GK, Pirie K, Beral V, Green J, Spencer E, Bull D (2007). Cancer incidence and mortality in relation to body mass index in the Million Women Study: cohort study. BMJ.

[11] Giovannucci E (2007). Metabolic syndrome, hyperinsulinemia, and colon cancer: a review. Am J Clin Nutr.

[12] Parkin E, O'Reilly DA, Sherlock DJ, Manoharan P, Renehan AG (2014). Excess adiposity and survival in patients with colorectal cancer: a systematic review. Obes Rev.

[13] Mantovani A, Allavena P, Sica A, Balkwill F (2008). Cancer-related inflammation. Nature.

[14] Xie Q, Matsunaga S, Niimi S, Ogawa S, Tokuyasu K, Sakakibara Y, Machida S (2004). Human lectin-like oxidized low-density lipoprotein receptor-1 functions as a dimer in living cells. Dna Cell Biol.

[15] Hirsch HA, Iliopoulos D, Joshi A, Zhang Y, Jaeger SA, Bulyk M, Tsichlis PN, Shirley LX, Struhl K (2010). A transcriptional signature and common gene networks link cancer with lipid metabolism and diverse human diseases. Cancer Cell.

[16] Yasumoto K, Hanagiri T, Takenoyama M (2009). Lung cancer-associated tumor antigens and the present status of immunotherapy against non-small-cell lung cancer. Gen Thorac Cardiovasc Surg.

[17] Zielinski C, Knapp S, Mascaux C, Hirsch F (2013). Rationale for targeting the immune system through checkpoint molecule blockade in the treatment of non-small-cell lung cancer. Ann Oncol.

[18] Hanahan D, Weinberg RA (2011). Hallmarks of cancer: the next generation. Cell.

[19] Amling CL, Riffenburgh RH, Sun L, Moul JW, Lance RS, Kusuda L, Sexton WJ, Soderdahl DW, Donahue TF, Foley JP, Chung AK, McLeod DG (2004). Pathologic variables and recurrence rates as related to obesity and race in men with prostate cancer undergoing radical prostatectomy. J Clin Oncol.

[20] Freedland SJ, Aronson WJ, Kane CJ, Presti JJ, Amling CL, Elashoff D, Terris MK (2004). Impact of obesity on biochemical control after radical prostatectomy for clinically localized prostate cancer: a report by the Shared Equal Access Regional Cancer Hospital database study group. J Clin Oncol.

[21] van Roermund JG, Kok DE, Wildhagen MF, Kiemeney LA, Struik F, Sloot S, van Oort IM, Hulsbergen-van DKC, van Leenders GJ, Bangma CH, Witjes JA (2009). Body mass index as a prognostic marker for biochemical recurrence in Dutch men treated with radical prostatectomy. Bju Int.

[22] Renehan AG, Soerjomataram I, Tyson M, Egger M, Zwahlen M, Coebergh JW, Buchan I (2010). Incident cancer burden attributable to excess body mass index in 30 European countries. Int J Cancer.

[23] Renehan AG, Tyson M, Egger M, Heller RF, Zwahlen M (2008). Body-mass index and incidence of cancer: a systematic review and meta-analysis of prospective observational studies. Lancet.

[24] De Pergola G, Silvestris F (2013). Obesity as a major risk factor for cancer. J Obes.

[25] Brunner AM, Sadrzadeh H, Feng Y, Drapkin BJ, Ballen KK, Attar EC, Amrein PC, McAfee SL, Chen YB, Neuberg DS, Fathi AT (2013). Association between baseline body mass index and overall survival among patients over age 60 with acute myeloid leukemia. Am J Hematol.

[26] Waki K, Yamada T, Yoshiyama K, Terazaki Y, Sakamoto S, Matsueda S, Komatsu N, Sugawara S, Takamori S, Itoh K, Yamada A (2014). PD-1 expression on peripheral blood T-cell subsets correlates with prognosis in non-small cell lung cancer. Cancer Sci.

[27] Samanic C, Gridley G, Chow WH, Lubin J, Hoover RN, Fraumeni JJ (2004). Obesity and cancer risk among white and black United States veterans. Cancer Causes Control.

[28] Wolk A, Gridley G, Svensson M, Nyren O, McLaughlin JK, Fraumeni JF, Adam HO (2001). A prospective study of obesity and cancer risk (Sweden). Cancer Causes Control.

[29] Yoshimoto S, Loo TM, Atarashi K, Kanda H, Sato S, Oyadomari S, Iwakura Y, Oshima K, Morita H, Hattori M, Honda K, Ishikawa Y, Hara E, Ohtani N (2013). Obesity-induced gut microbial metabolite promotes liver cancer through senescence secretome. Nature.

[30] Renehan AG (2009). Bariatric surgery, weight reduction, and cancer prevention. Lancet Oncol.

[31] Kempf E, Hirsch P, Labopin M, Viguie F, Isnard F, Tang R, Marzac C, Marie JP, Mohty M, Legrand O (2014). Prognosis of body mass index and chemotherapy dose capping in acute myeloid leukaemia. Leuk Res.

[32] Medeiros BC, Othus M, Estey EH, Fang M, Appelbaum FR (2012). Impact of body-mass index on the outcome of adult patients with acute myeloid leukemia. Haematologica.

[33] Bhindi B, Trottier G, Elharram M, Fernandes KA, Lockwood G, Toi A, Hersey KM, Finelli A, Evans A, van der Kwast TH, Fleshner NE (2012). Measurement of peri-prostatic fat thickness using transrectal ultrasonography (TRUS): a new risk factor for prostate cancer. Bju Int.

[34] Larsson SC, Wolk A (2007). Overweight, obesity and risk of liver cancer: a meta-analysis of cohort studies. Br J Cancer.

[35] Wiseman M (2008). The second World Cancer Research Fund/American Institute for Cancer Research expert report. Food, nutrition, physical activity, and the prevention of cancer: a global perspective. Proc Nutr Soc.

[36] Berrington DGA, Sweetland S, Spencer E (2003). A meta-analysis of obesity and the risk of pancreatic cancer. Br J Cancer.

[37] Moghaddam AA, Woodward M, Huxley R (2007). Obesity and risk of colorectal cancer: a meta-analysis of 31 studies with 70,000 events. Cancer Epidemiol Biomarkers Prev.

[38] Larsson SC, Wolk A (2007). Obesity and colon and rectal cancer risk: a meta-analysis of prospective studies. Am J Clin Nutr.

[39] Kubo A, Corley DA (2006). Body mass index and adenocarcinomas of the esophagus or gastric cardia: a systematic review and meta-analysis. Cancer Epidemiol Biomarkers Prev.

[40] Kathiresan S, Melander O, Guiducci C, Surti A, Burtt NP, Rieder MJ, Cooper GM, Roos C, Voight BF, Havulinna AS, Wahlstrand B, Hedner T, Corella D, Tai ES, Ordovas JM, Berglund G (2008). Six new loci associated with blood low-density lipoprotein cholesterol, high-density lipoprotein cholesterol or triglycerides in humans. Nat Genet.

[41] Yang Y, Morin PJ, Han WF, Chen T, Bornman DM, Gabrielson EW, Pizer ES (2003). Regulation of fatty acid synthase expression in breast cancer by sterol regulatory element binding protein-1c. Exp Cell Res.

[42] Shah US, Dhir R, Gollin SM, Chandran UR, Lewis D, Acquafondata M, Pflug BR (2006). Fatty acid synthase gene overexpression and copy number gain in prostate adenocarcinoma. Hum Pathol.

[43] Migita T, Ruiz S, Fornari A, Fiorentino M, Priolo C, Zadra G, Inazuka F, Grisanzio C, Palescandolo E, Shin E, Fiore C, Xie W, Kung AL, Febbo PG, Subramanian A, Mucci L (2009). Fatty acid synthase: a metabolic enzyme and candidate oncogene in prostate cancer. J Natl Cancer Inst.

[44] Baron A, Migita T, Tang D, Loda M (2004). Fatty acid synthase: a metabolic oncogene in prostate cancer?. J Cell Biochem.

[45] Alo’ PL, Visca P, Marci A, Mangoni A, Botti C, Di Tondo U (1996). Expression of fatty acid synthase (FAS) as a predictor of recurrence in stage I breast carcinoma patients. Cancer.

[46] Petersen I, Warth A (2014). [Lung cancer. Developments, concepts and preview of the new WHO classification]. Pathologe.

