# Supplementary material for: Combination of body mass index and oxidized low density lipoprotein receptor 1 in prognosis prediction of patients with squamous non-small cell lung cancer
Source: Oncotarget. 2015 May 27;6(26):22072–80. doi: 10.18632/oncotarget.4299 (PMC4673147; doi:10.18632/oncotarget.4299)
Supplement: Supplementary file 1 [file oncotarget-06-22072-s001.pdf]

Combination of body mass index and oxidized low density lipoprotein receptor 1 in prognosis prediction of patients with squamous non-small cell lung cancer

Supplementary Material

Supplemental Table S1: Univariate and multivariable analysis with prognostic role of OLR1 and BMI in PFS for patients with squamous non-small cell lung cancer

Variables	Univariate analysis			Multivariable analysis		
	HR	95%CI	P value	HR	95%CI	P value
BMI	1.635	1.151-2.138	0.022	1.145	1.0841.432	0.019
OLR1	3.592	2.544-5.072	<0.001	3.541	2.514-4.988	<0.001
